# SERS-Based Local Field Enhancement in Biosensing Applications

**DOI:** 10.3390/molecules30010105

**Published:** 2024-12-30

**Authors:** Yangdong Xie, Jiling Xu, Danyang Shao, Yuxin Liu, Xuzhou Qu, Songtao Hu, Biao Dong

**Affiliations:** State Key Laboratory on Integrated Optoelectronics, College of Electronic Science and Engineering, Jilin University, Changchun 130012, China; ydxie22@mails.jlu.edu.cn (Y.X.); jlxu23@mails.jlu.edu.cn (J.X.); shaody1922@mails.jlu.edu.cn (D.S.); yxliu1922@mails.jlu.edu.cn (Y.L.); quxz1922@mails.jlu.edu.cn (X.Q.); hust21@mails.jlu.edu.cn (S.H.)

**Keywords:** SERS, biological detection, composite nanoparticles, plasma

## Abstract

Surface-enhanced Raman scattering (SERS) stands out as a highly effective molecular identification technique, renowned for its exceptional sensitivity, specificity, and non-destructive nature. It has become a main technology in various sectors, including biological detection and imaging, environmental monitoring, and food safety. With the development of material science and the expansion of application fields, SERS substrate materials have also undergone significant changes: from precious metals to semiconductors, from single crystals to composite particles, from rigid to flexible substrates, and from two-dimensional to three-dimensional structures. This report delves into the advancements of the three latest types of SERS substrates: colloidal, chip-based, and tip-enhanced Raman spectroscopy. It explores the design principles, distinctive functionalities, and factors that influence SERS signal enhancement within various SERS-active nanomaterials. Furthermore, it provides an outlook on the future challenges and trends in the field. The insights presented are expected to aid researchers in the development and fabrication of SERS substrates that are not only more efficient but also more cost-effective. This progress is crucial for the multifunctionalization of SERS substrates and for their successful implementation in real-world applications.

## 1. Introduction

The molecular level detection of biomolecules, biological metabolites, and other substances can be used to demonstrate the physiological reaction process of an organism, which is one of the means to evaluate the level of cellular metabolism and the effect of clinical treatment [1]. The detection of biomolecules and biological metabolites has become an important part of clinical medicine, environmental management, cancer prevention, food safety, and other fields. Therefore, an efficient, rapid, sensitive, micro-quantitative, and molecular-specific sensing method is necessary to detect the biological information contained in trace liquid [2,3].

**Figure 1:**
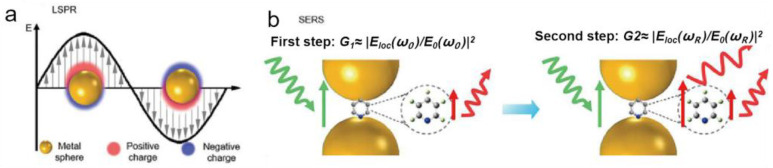
(**a**) Schematic representation of local surface plasmon resonance (LSPR), (**b**) schematic representation of the electromagnetic enhancement mechanism of surface-enhanced Raman scattering (SERS) [18].

Surface-enhanced Raman scattering (SERS), as a new ultrasensitive detection method, has the advantages of simple operation, short time consumption, high sensitivity and specificity [4,5]. Since the first observation of SERS by Fleischmann et al. in 1974 by adsorping pyridine molecules on a rough silver electrode, Raman analytical methods have been widely used for trace chemical analysis [6]. Electromagnetic enhancement (EM) and chemical enhancement (CM) are currently recognized as the two main mechanisms [7,8]. Among them, electromagnetic field enhancement is the main contribution of SERS enhancement, which can reach 10^10^–10^12^ orders of magnitude in single-molecule SERS, while chemical enhancement is basically maintained at 10^1^–10^3^ orders of magnitude [9]. Therefore, EM plays a major role in SERS detection. Such localized regions with extremely strong electromagnetic fields are often referred to as “hot spots” (LSPR) due to the local surface plasmon resonance (LSPR) effect [10]. As shown in Figure 1, when the frequency of the excitation light matches the surface plasmon resonance frequency of the metal nanostructure, the electromagnetic field intensity increases significantly [11,12].

As a rapid, real-time, highly sensitive, and specific detection technology, SERS can be used in food safety detection of pesticide residues, and pest and disease effects; in biomedical detection, such as circulating tumor cells and exosomes; in terms of environmental detection, such as air pollutants and water pollutants; and in other medical and disease diagnosis, with a very wide range of applications [13]. At the same time, SERS can also characterize the process of some experimental reactions through its unique optical generation process [14]. For example, the process of photocatalytic reactions is shown [15], and biomolecules are imaged to explore their components [16]. Therefore, SERS technology has been widely concerned by researchers.

The main parameters that affect SERS performance include hot spot density, enhancement factor, detection limit, uniformity, resolution, Raman cross section, and reuse rate [17]. Based on the existing research results, researchers hope to achieve lower cost and efficient SERS enhancement strategies, such as using semiconductors or even polymers instead of plasma as the electromagnetic core, directly recognizing the functional groups on the bacterial cell surface for label-less biological detection, and designing more uniform, ordered, and highly sensitive substrates for single-molecule detection and distribution statistics, breaking the optical diffraction limit, pursuing smaller-scale tip Raman enhancement, etc.

**Figure 2:**
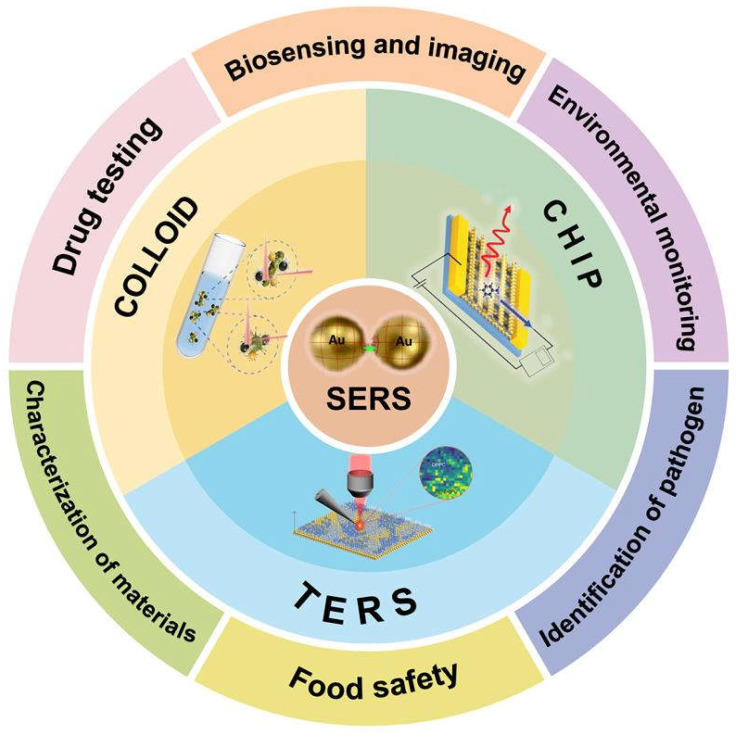
Schematic of latest progress of SERS in nanostructures, substrates, and TERS.

Therefore, in recent years, a lot of work has been done on SERS, including biochemical detection, environmental analysis, food safety, single-molecule identification statistics, real-time chemical reaction detection, and so on. Although the detection accuracy, range, and function vary from work to work, these SERS works cannot be separated from the basic structure, that is, the nanoparticles providing electromagnetic enhancement and the substrate carrying particles. Therefore, we hope to do a detailed summary by the morphology of nanoparticles and the type of substrate. For example, nanoparticles from isotropic to anisotropic, from single crystal to composite; substrate types range from 2D to 3D and from non-optical to optical. We expect to show the latest SERS work in recent years through a structural division (Figure 2), from nanoparticles in the electromagnetic center to various substrates with different functions, and then to TERS derived from SERS, so that researchers can more quickly consult and understand these works.

## 2. Nanostructures (Colloids) in SERS Sensors

Nanoparticles are the core structures in SERS sensors, and the electromagnetic (EM) enhancement provided by nanoparticles is the main part of Raman enhancement. Since SERS hotspots mostly exist at the tip, gap, and angle of plasmons [19], it is meaningful to modify the structure of noble metal nanomaterials. Researchers have also designed nanoparticles with different shapes, such as nanorods, nanowires [20,21,22], nanodisks [23,24,25], nanospikes, nanostars [26,27,28], and others. In addition, using core–shell or MOF structure to combine multifunctional materials such as polymers, semiconductors, and oxides with noble metal nanoparticles can overcome the problems of easy agglomeration and oxidation of single crystalline nanoparticles. The SERS signal can be maximized to enrich its application in biological detection [29].

### 2.1. Single Crystalline Nanoparticles

The SERS sensitivity of plasmonic nanoparticles is affected by their size and morphology. The plasmas in the earliest SERS sensors are spherical or quasi-spherical. Now, plasmonic nanoparticles that study surface anisotropy have better SERS sensitivity. More attention has been paid to nanorods, nanospines, nanopores, and other nanostructures that can generate higher hot spots [30].

#### 2.1.1. Nanorods and Nanowires

Gold nanorods (Au NRs) are the most commonly used nanostructures besides nanospheres due to well-established synthesis methods and fairly high yields. The absorption peak can be precisely tuned by adjusting the aspect ratio of the nanorod, and the variable absorption peak enables it to correspond to a variety of Raman laser wavelengths [31].

**Figure 3:**
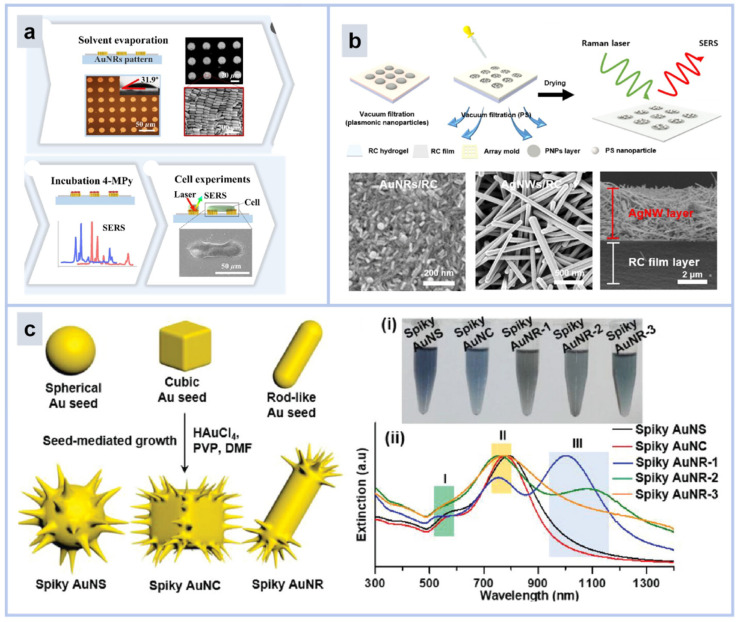
(**a**) Schematic diagram of gold nanorods used to detect bacteria [32]. (**b**) Schematic diagram of silver nanowires used for detection of Nano-plastics [33]. (**c**) Schematic diagram of synthesis of gold nanospikes with different shapes. (i) Optical photograph and (ii) optical extinction spectra of spiky Au NPs in water synthesized with various shaped Au seeds. I is the intrinsic absorption peak of the plasma core, II is the absorption peak due to the growth spikes, and III is the second absorption peak of the gold nanorods [34].

Dabum Kim et al. reported a cell monitoring substrate based on gold nanorods [32]. As shown in Figure 3a, using the wettability differential interface method, Au NRs were neatly self-assembled into micro-circles, and the micro-circles were neatly arranged in arrays. This structure can provide rough terrain for cell arrangement and facilitate cell adhesion. In addition, Au NRs can also provide a strong SERS effect for cell biological detection. Au NRs micropatterns guided cell contact and lying down, and caused morphological changes in the cells, and the pH changes during cell alignment were also able to be obtained by SERS spectroscopy, in which the pH near the cytoplasm was lower than that of the nucleus, revealing the heterogeneity of the extracellular microenvironment. 

Given the dual functions of cell capture and SERS enhancement of Au NRs micropatterns, this integrated array platform can be used to demonstrate the interaction between cells and interfaces when they are in contact, providing certain assistance to the regulatory and response mechanisms of cells. However, it should be noted that the changes of cell morphology caused by this microstructure may be unfavorable to the cells, so the feedback of apoptosis morphology is obtained. Secondly, the arrangement of gold nanorods array does not have a sufficiently sensitive SERS signal, and the information obtained is only the part at the bottom of the cell in contact with the substrate, so it is difficult to show the SERS information inside the cell or even the whole cell. Therefore, a SERS substrate with a three-dimensional form is desirable when facing large-structure biological analytes such as cellular bacteria. Furthermore, the application of SERS in large-scale bioanalyte detection needs to be further explored.

In nanowires, a variant of nanorods, the SERS enhancement factor of Ag NPs is typically one to two orders of magnitude higher than that of Au NPs. As shown in Figure 3b, Youngho Jeon et al. prepared a SERS sensor for silver nanowires [33]. Through a simple vacuum-assisted filtration method using a silicon mask, a surface-enhanced Raman scattering (SERS) active array substrate was constructed using regenerated cellulose (RC) and plasmonic nanoparticles (Au NR and Ag NW) for rapid nanoplastic detection. Experimental results showed that Ag NW was superior to Au NR in sensitivity. Among them, the enhancement factor (EF) of Ag NWs is 1.8 × 10^7^, and the detection limit of Ag NWs/RC films for PS nanoplastics is 0.1 mg/mL. It provides an effective tool for the detection of nanoplastic pollutants in water.

Similarly, Pang et al. also proposed a self-assembled thin film composed of Ag NWs [35]. They arranged self-assembly through the water–oil–gas three-phase interface, and realized a highly neat arrangement of Ag NWs. This neat arrangement ensures it has a considerable laser matching degree. It exhibits an enhancement factor of 6.12 × 10^11^ and a detection limit of up to 1.0 × 10^−16^ M for R6G. This highly sensitive, self-assembled SERS substrate has a wide range of detection application scenarios.

#### 2.1.2. Nano Spikes

It has been demonstrated that anisotropic nanoparticles perform better in SERS, because more tips and gaps will generate more hot spots during nanoparticle alignment. Therefore, nanospikes and nanostars have attracted much attention.

Jiang et al. conducted a comprehensive study of the plasmonic and photothermal properties of spiked-gold nanoparticles, examining the changes in their core shape that gradually form spikes, and the effect of this change on SERS performance, including nanospheres, nanocubes, and nanorods (Figure 3c) [34]. The results show that the central core shape of spiked Au NPs has a significant effect on their SERS and photothermal properties, and that spiked Au NPs with spherical or cubic cores are much superior for near-infrared performance. This is due to the fact that the anisotropy tendency of the sphere and cube intensifies more obviously during the spiking process. The spike-based SERS probes based on gold nanoparticles demonstrated excellent ability for live-cell SERS imaging and tumor dynamic visualization in a 4T1 mammary tumor mouse model after intravenous injection. Spiking SERS probes based on gold nanoparticles are potentially useful in effective SERS-guided cancer hyperthermia.

#### 2.1.3. Nanopores

**Figure 4:**
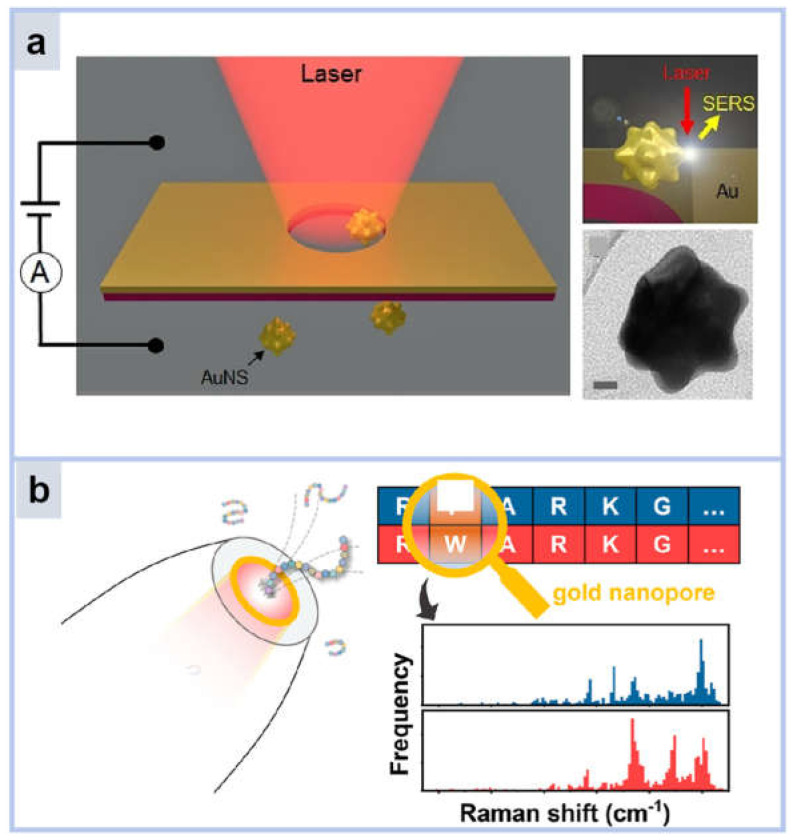
(**a**) Schematic representation of gold nanoparticles trapped in plasmonic nanopores. (**b**) Schematic representation of gold plasmonic nanopores to detect peptides and their single point mutated variants.

Nanopore refers to the pore structure with a diameter of nanometer, which can be used for molecular screening and separation. Single-molecule level detection nanopore has a strong hot spot, by monitoring the change of SERS signal when a single molecule passes through the nanopore [36]. Especially, the SERS signal changes very strongly when the molecules bind to the metal nanoparticles and pass through the nanopores. As shown in Figure 4a, Huang et al. trapped gold nanoparticles in plasmonic nanopores [37] to generate single SERS hotspots for single-molecule detection of two similar peptides, vasopressin (VAS) and oxytocin (OXT), and 10 different amino acids that make up the two peptides. This method is able to distinguish VAS from OXT by Raman spectra of amino acid residues, including non-aromatic residues. As shown in Figure 4b, a large number of SM-SERS spectra were obtained by high-throughput spectral acquisition, which laid the foundation for the statistical analysis of single-molecule spectra. Then, a method for SM-SERS spectral data processing based on Boolean operations was proposed and applied to reveal statistically significant features of peptide structures, despite the presence of spectral fluctuations. This approach also makes it possible to study the effects of single-point mutations on peptide structure at the single-molecule level.

### 2.2. Core–Shell Nanoparticles

Single crystalline nanoparticles are easy to oxidize, easy to agglomerate, and difficult to control the arrangement and gap, which limits their application in SERS sensing [38]. Core–shell nanoparticles are composed of a core and a shell made of different materials. Thus, the combination of different properties of different materials leads to several new properties of core–shell materials, thereby overcoming the shortcomings of single crystalline nanoparticles and expanding their applications in electronics, optics, magnetism, and catalysis [39]. This kind of method can be used to detect RNA, DNA, and other biological molecules, but also can be used to detect cells, bacteria, and other biological individuals. Its advantages are high sensitivity and low detection limit, which can achieve the detection level of pM/L and even fM/L [40,41]. In addition, by means of label adsorption, it can avoid the disadvantage of SERS near-field enhancement, and cannot detect large biological individuals.

#### 2.2.1. SERS Sensor with Metal Core–Shell Structure

**Figure 5:**
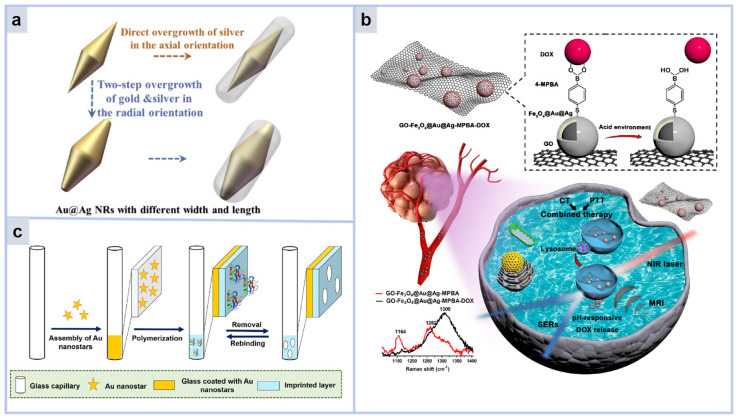
(**a**) Schematic representation of the core–shell Au@Ag NRs with controllable width and length [46]. (**b**) Schematic of graphene oxide (GO) nanocomposites deposited by Fe_3_O_4_@Au@Ag nanoparticles [56]. (**c**) Schematic representation of capillary glass SERS sensor for label-free recognition of specific proteins [57].

In recent years, Au/Ag has been linked as a bimetallic method such as core/shell or alloy, and its physical and chemical properties have been fully explored through SERS research, which has great potential to be used to detect biomolecules [42]. Such Au/Ag core–shell nanoparticles can adjust the electronic structure and properties of the metal usually with adjustable surface plasmon resonance (SPR) properties, outstanding enhancement and stability [43,44,45]. Lin et al. produced core–shell Au@Ag nanorods (NRs) with controllable width and length as excellent SERS substrates [46]. Gold Nanobipyramids (Au NBPs) of three different widths were prepared by overgrowth along the radial direction (Figure 5a), and core–shell Au@Ag NRs with different lengths and widths were successfully obtained by deposing silver in the axial direction with Au NBPs as seeds. The systematic examination of the synthesized Au@Ag NRs’ structure, light absorption characteristics, and SERS efficiency has been conducted. The broadly tuned UV-vis absorption range of Au@Ag NR can reach the near infrared (NR) region. For Au@Ag NRs with wide width and long length, the EF was as high as 6.13 × 10^5^.

In addition, the combination of magnetic materials with gold or silver shells makes them suitable for biological separation and detection [47,48,49]. For example, Figure 5b, Huang et al. proposed a new pH-responsive nanosystem [49]. Using 4-mercaptophenylboronic acid as a Raman signal reporting molecule, Fe_3_O_4_@Au@Ag NPs with high activity and stability were deposited on the surface of graphene oxide, forming the GO-Fe_3_O_4_@Au@Ag-MPBA Raman probe. Then, doxorubicin was linked to 4-MPBA through an ester bond. Upon entering the tumor, the acidic environment within the tumor would cause the ester bond to break, releasing DOX and restoring the 4-MPBA SERS signal. Finally, the Raman signal intensity of 4-MPBA was used to determine the release of DOX. This work has great potential in the real-time detection and evaluation of cancer therapy.

However, the enrichment and movement of magnetic particles in vivo, and the activation of these particles in vivo by infrared wavelength excitation light, remain unstable for Raman scattering imaging. In this respect, it is still expected to have more specific identification and enrichment capabilities.

#### 2.2.2. SERS Sensor with Non-Metallic Core–Shell Structure

As the isolation layer of nanoparticles, the non-metallic shell is used to protect the internal noble metal core, avoid direct contact between probe molecules and the environment, reduce background interference in liquid biopsy, and avoid its oxidation and agglomeration [39]. In addition, the protective shell not only prevents the aggregation of nanoparticles, but also regulates the spacing and porosity of nanoparticles and has good biocompatibility. Therefore, it is suitable for biological analysis. In such studies, silica [50,51,52,53], polymers [54,55], or other biomolecules are commonly used as shell materials.

Silica possesses several intrinsic benefits, including resistance to chemical reactions, compatibility with biological systems, and clarity in optical applications. Due to these attributes, silica emerges as a viable material for use in the optical industry, biotechnology, and various other sectors. Jessi E.S. Hoeven et al. introduced a silica-coated gold nanorod assembled into spherical aggregates called superparticles [56], in which the plasma coupling and mass transport are under control by the thickness and porosity of the silica shell. Using crystal violet as the probe molecule, the aggregated superparticles successfully amplify the Raman signal. Moreover, as the thickness of the silicon shell decreases, the Raman signal in the superparticle is further enhanced. This work successfully verified the role of non-metallic shell in the gap regulation of SERS and demonstrated the interference of non-metallic shell on the Raman signal intensity. It provides experience for exploring the balance between SERS intensity and material properties in the bonding process of nonmetallic shell and plasmonic nanoparticles.

Besides silica, a variety of non-toxic and biocompatible polymers have garnered significant interest. These polymers offer several benefits over silica, such as being non-toxic, featuring a robust and pliable outer layer, exhibiting minimal adsorption, and displaying superior bioavailability and pharmacokinetic characteristics [58]. Therefore, some researchers have paid more attention to the biological applications of polymer shells. For example, Maryam Arabi’s group designed a capillary glass SERS sensor that can recognize specific proteins without labels [57] (Figure 5c). It comprises a core layer of gold nanoparticles designed to amplify signals and an outer layer of polydopamine imprinting that serves as the identifying component. The imprinted layer is used to selectively capture the target protein and to allow the Raman indicator to enter the signal enhanced substrate layer. Specific protein recognition would fill the imprinted cavity and block access of the Raman indicator. The amount of captured protein can be reflected by the signal reduction of the Raman activity indicator molecule. The capillary sensor performed specific and reproducible detection of trypsin in the biological samples received at levels as low as 4.1 × 10^−3^ μg/L without sample preparation.

In addition, the combination of non-metallic shell and noble metal nanoparticles can expand a variety of functions. For example, cerium oxide shell and titanium oxide shell of gold nanorods are commonly used as catalytic enzymes in enzyme cascade reactions and SERS labels [59,60]. And nanoparticles of molecular shells including DNA and proteins are commonly used as SERS tags for bioanalysis and bioimaging [61,62].

During the modification of these multifunctional nonmetallic shells, the thicker nonmetallic shells may affect the intensity of SERS. Thicker shells will bring more functions that vary greatly, like increased adsorption, enrichment, specificity of performance, avoiding agglomeration caused by the analysis results, etc. However, these non-metallic shells can hinder the adsorption of analytes with nanoparticles and reduce the Raman scattering cross section to a certain extent. Therefore, researchers must balance the sensitivity of SERS with the functionality of the nonmetallic shell to achieve the functionality of the nonmetallic shell without affecting the SERS spectral intensity.

### 2.3. SERS Sensor with MOF Structure

Metal–organic frameworks (MOFs) have an infinite lattice and ordered periodic grids. The organic ligand that serves as the framework and the metal cation at the node are linked by a coordination bond of moderate strength [63]. Therefore, the unique structure of MOF leads to its special physical and chemical properties: ① a very high specific surface area, which can quickly adsorb and enrich signal molecules; ② uniform micro/nanopores with good molecular size selectivity; ③ quite good chemical and thermodynamic stability; and ④ charge transfer of a MOF can promote CM enhancement. In recent years, more and more studies have applied a MOF to SERS, and based on its good physicochemical properties, it has been used to solve SERS detection that is difficult to achieve with conventional plasma substrates.

**Figure 6:**
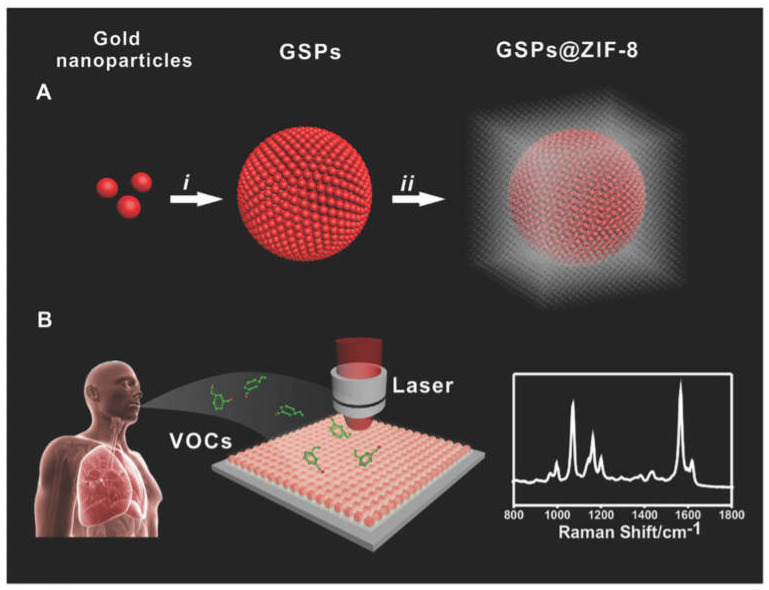
GSPs@ZIF-8 schematic diagram of gas biomarkers for selective detection of lung malignancies [67]. (**A**) The synthesis process of GSPs@ZIF-8. (**B**) Forms of Raman detection for VOCs.

For example, PNP-MOF substrates can not only identify normal biomarkers but also enable SERS detection of VOC biomarkers through the synergistic interaction of MOF (molecular adsorption) and PNPs (EM) [64,65,66]. Qiao et al. reported a ZIF-8 layer coated with gold superparticles (GSPs) substrate (GSPs@ZIF-8) [67], which can selectively detect biomarkers of lung malignancies at the ppb level in Figure 6. The introduction of ZIF-8 enhanced the absorption of gas molecules, slowing the flow rate of gas biomarkers. The rapid decline of the electromagnetic field near the GSP surface is mitigated. Moreover, this potent platform exhibits the capability to selectively detect minute quantities of gaseous cancer indicators within a gaseous blend, and it overcomes the long-standing limitations of detecting gaseous molecules on solid substrates. Therefore, the introduction of suitable MOF for rational construction of effective SERS substrates, especially PNP-MOFs with adjustable pore size, will help to address some challenges in SERS applications, such as improving enrichment capacity, enhancing molecular selectivity, and increasing synthetic diversity.

Notably, SERS enhancement has also been found in some MOFs such as MIL-100 and ZIF-8 [68,69,70,71,72], and such MOFs can function as active SERS substrates in the absence of noble metals. Unlike the common electromagnetic enhancement (EM), the major contribution of this class of MOF to SERS originates from the charge transfer mechanism (CT)-guided chemical enhancement (CM). Zhao Zhenxia’s team investigated the possibility of MIL-100 (Fe) as a substrate for SERS in the absence of noble metals, and verified that its enhancement factor EF can reach 105 under the action of chemical enhancement (CM). Different gases of lung cancer were detected, and the detection limit was ppm, which showed its great potential as SERS substrate [68].

## 3. The Substrate in the SERS Sensor

In order to avoid the problem that the aggregation and dispersion state of colloidal SERS sensor colloidal particles may affect the stability and reproducibility of SERS signal [73]. A chip-type SERS sensor was fabricated by arranging noble metal nanoparticles on the substrate. This type of sensor has a uniform SERS active site, and the standard deviation of the signal is within 10%. Through the self-alignment of metal nanoparticles, the signal is uniform, the detection is fast, and the repeatability and stability are good [74,75].

In addition to the self-assembly method to directly align the electromagnetic enhancing cores of noble metals on a range of smooth substrates such as silicon or glass substrates, a range of 2D or tri-substrate substrates such as semiconductors, polymers, and microfluidics have been developed to extend the functionalities of SERS.

### 3.1. Self-Assembly of Nanoparticles

The self-assembly method is the most commonly used method for fabricating SERS sensors, in which noble metal nanoparticles are uniformly arranged on smooth silicon or glass. The size and shape of the nanoparticles were modulated to increase hot spots as much as possible. Such self-assembly methods usually include two-liquid level method [76,77], three-liquid level method [78,79], Langmuir–Blodgett [80,81], electrostatic force self-assembly [78,82], and other methods.

**Figure 7:**
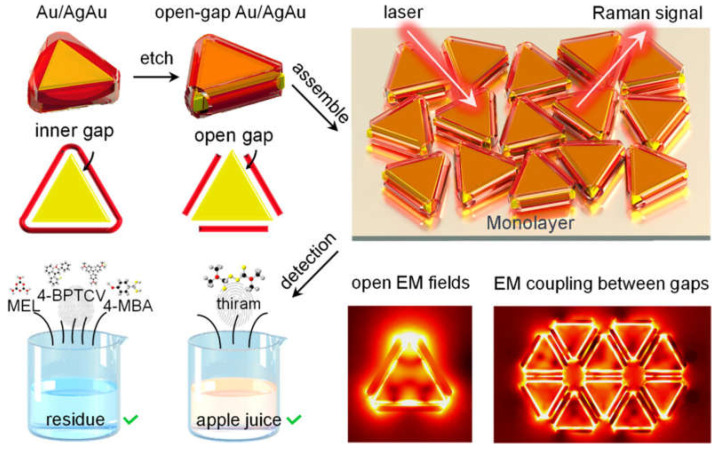
Schematic diagram of uniform monolayer arrangement of Au-Ag core–shell nanoparticles at the mouth of the triangle [83].

For example, Ma Liang’s research group prepared a highly uniform Au/AgAu monolayer SERS substrate by self-assembly method [83], which has rich nanogaps and huge electromagnetic enhancement effects through a triangle and a single layer of Au-silver core–shell nanoparticles with special openings in the triangle in Figure 7. The monolayer material showed good signal stability, uniformity, and reproducibility. The analytical enhancement factor of crystal violet detection was 2.12 × 10^8^, and the relative standard deviation was 4.65%.

Similarly, Tian et al. uniformly arranged petal-shaped Ag NPs to form a monolayer film substrate through the three-phase interface self-assembly method [84]. This uniform assembly method largely solved the agglomeration problem of nanoparticles, even for rough petal-shaped Ag NPs thin film substrates. The relative standard deviation (RSD) was 6.5% and the enhancement factor was 2.7 × 10^7^.

These self-assembly methods are fast and convenient, and can obtain good properties, which are common methods in the rapid preparation of SERS substrates. However, because most of the self-assembly methods are uniform monolayer films, and the hotspot density is limited by the Cartesian surface, even high-hotspot nanoparticles such as nanostars and spines are difficult to achieve high detection performance.

### 3.2. 2D Substrate Materials

A range of two-dimensional (2D) materials have emerged as cost-effective SERS substrates due to their straightforward synthesis, remarkable optical characteristics, and favorable biocompatibility. Furthermore, these 2D materials possess distinctive physicochemical traits, including adjustable electronic configurations, elevated mobility of charge carriers, resistance to chemical reactions, and pliability [85]. Consequently, various two-dimensional materials, including hexagonal boron nitride (h-BN), two-dimensional perovskite, graphitic carbon nitride (g-C3N4), transition metal dichalcogenides (TMDs), black phosphorus (BP), and transition metal carbides and nitrides (MXenes) have been explored for a range of applications [86,87,88,89].

#### 3.2.1. Graphene, h-BN, g-C_3_N_4_

**Figure 8:**
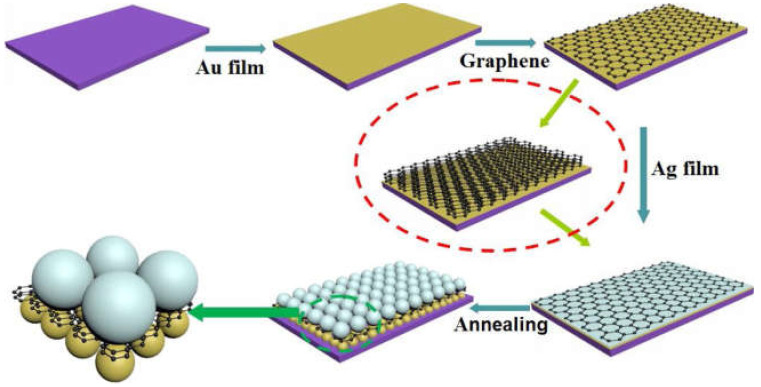
Schematic diagram of Ag NPs/graphene@AuNPs sensor with 3D hot spot and adjustable nanogap [90].

Graphene, due to its special physicochemical properties of high specific surface area, high electron mobility, high transparency, and high electron density, has great potential in SERS [87]. Zhang Chao et al. proposed an Ag NPs/graphene@Au NPs system with 3D hot spots and adjustable nanogaps [90]. In Figure 8, graphene layer is altered by a simple method. R6G and CV were used as probe molecules with detection limits of 10^−11^ M and 10^−12^ M, respectively. For ecological environment detection, the prepared Ag NPs/graphene@Au NPs substrates were used to detect malachite green (MG) in seawater samples. The detection limit can reach 10^−11^, based on the good charge transport ability of graphene and the electromagnetic enhancement ability of Ag NPs/Au NPs. In this work, an excellent detection limit was obtained and analytes could be detected in a complex system such as seawater.

In addition, h-BN and g-C_3_N_4_ have also been applied to the 2D substrates of SERS, both of which have a graphite-like layered structure. Ling et al. investigated the Raman enhancement effect of h-BN using CuPc molecules as probes. Unlike graphene, the Raman intensity of CuPc does not depend on the h-BN layer due to the different charge transfer process. In h-BN, the N atom tends to confine more electron pairs from the sp2 hybrid B-N bond due to its higher electronegativities, thus inducing enhanced interfacial dipoles between h-BN and the molecule. Dipole interactions indicate that h-BN is a superior substrate in terms of uniformity [91]. These graphene or graphene-like 2D materials, due to their good conductivity and high surface area, can enhance CM enhancement in SERS by facilitating charge transfer in addition to assisting the EM enhancement of nanoparticles. Although the improvement brought by such 2D substrates is obvious, due to the complexity of material preparation, the stacking of 2D materials during the substrate formation process will cause a decrease in homogeneity compared with the self-assembly method, and further breakthroughs in large-scale fabrication and recycling are still needed.

#### 3.2.2. Black Scales (BP)

**Figure 9:**
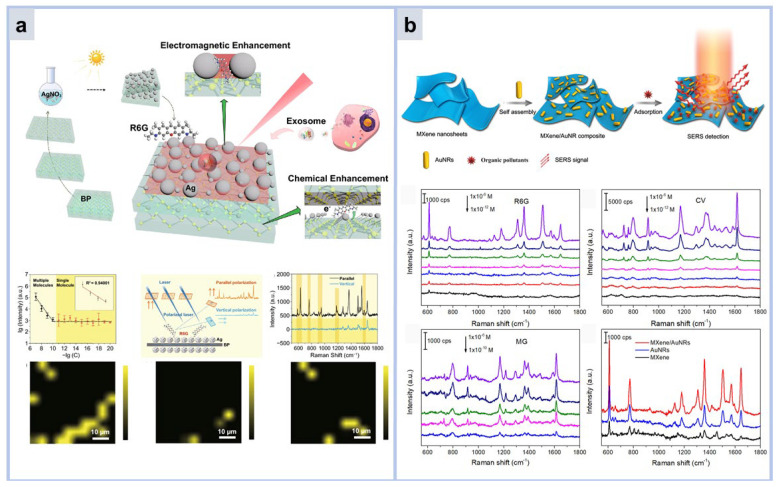
(**a**) Schematic representation of Ag/BP-NS substrate and Raman Mapping of R6G single-molecule detection [93]. (**b**) Schematic representation of SERS sensor with Mxene as substrate bearing gold rod, and their detection limits for R6G, CV, and MG, respectively [96].

BP have different crystal structures and properties, which may make them promising SERS substrates. Similar to graphite, 2D BP is a single-element material whose layers are stacked together by van der Waals forces [92]. For a monolayer BP, a P atom is bonded to three neighboring atoms, showing a ridge-like structure when viewed from the side and a honeycomb structure when viewed from the top view. Moreover, BP is non-invasive and non-toxic to biological tissues, ensuring its use as an effective SERS substrate for a wide range of biosensing. Lin et al. reported a specially engineered nanostructure, as shown in Figure 9a. Silver nanoparticles incorporated into multilayer black phosphorus nanosheets (Ag/BP-NS), produced via a novel photoreduction technique, function as SERS sensor. This SERS platform achieves a detection threshold for Rhodamine 6G (R6G) at 10^−20^ mol/L, facilitating the Raman mapping for single-molecule analysis. This performance is attributed to the synergistic enhancement from three resonances: molecular resonance, electromagnetic resonance, and photo-induced charge transfer resonance. Moreover, when integrated with machine learning algorithms, the Ag/BP-NS substrates can distinguish various tumor exosomes, which is crucial for cancer surveillance and early detection [93]. However, the chemical properties of black scales and Ag NPs themselves are not stable, and long-term exposure to air will oxidize and drastically reduce SERS intensity. Therefore, the preparation of long-term stable BP based SERS substrates to obtain the performance of single-molecule detection is still a problem to be solved.

#### 3.2.3. MXenes

The general formula for MXenes is M_n+1_X_n_Tx, where M represents the early transition metal, X is carbon and/or nitrogen, n represents the number of X layers, and T is the surface termination end (-O, -OH, -F, or -Cl). In MXenes, the X layer is covered by the M layer and Tx is bound to the outer layer of M; therefore, they may exhibit favorable properties such as high metallic conductivity and hydrophilicity, which enable them to be SERS substrates [94]. MXenes can electrostatically interact with noble metal nanoparticles, resulting in large-scale nanoparticle loading. E. Satheeshkumar et al. reported that Ag, Au, and Pd@MXene (Ti_3_C_2_Tx) hybrids, due to the combination of EM and CM, exhibit enhanced Raman signal to MB molecules [95]. Xie Hanhan et al. designed Ti_3_C_2_Tx/Au NRs for organic pesticide detection [96]. For Raman indicators like Rhodamine 6G (R6G), Crystal Violet (CV), and Malachite Green (MG), the sensitivity thresholds were 10^−12^, 10^−12^, and 10^−10^ M, respectively, accompanied by minimal relative standard deviations as depicted in Figure 9b. Building on these findings, the Raman detection of two common organic pesticides, thiram and diquat, was investigated. On the Ti_3_C_2_Tx/Au NRs platform, discernible Raman signals were observed at concentrations of 10^−10^ and 10^−8^ M for thiram and diquat, respectively.

These two-dimensional nanomaterials and their composites boast distinctive benefits, including facile synthesis, expansive specific surface areas, superior mechanical attributes, remarkable optical characteristics, and favorable biocompatibility, all of which facilitate their effective utilization in SERS enhancement. It also provides suitable solutions to solve the challenges of high cost of metal substrates, strong metal–adsorbate interaction, catalysis, and photobleaching. However, as mentioned before, both graphene and black scale are accompanied by the problems of stability and the need to optimize the preparation process. Moreover, many works have mentioned that CM enhancement has made a great contribution to the detection of two-dimensional substrates in SERS, and the exploration of this aspect still needs to be deepened.

### 3.3. 3D Substrate Materials

As research progresses, it is difficult for self-assembled nanoparticle substrates to meet higher sensitivity detection, and 2D substrates are similar to self-assembled substrates, where the maximum number of available SERS hotspots is limited by a single Cartesian plane [97]. The laser confocal volume in SERS instrument is a three-dimensional space, which means that these substrates do not make full use of the effective confocal volume, and in the limited space of the area to be tested, more hotspots cannot be added to enhance the signal intensity. Therefore, in addition to the regulation of precious metal nanoparticles, the substrate bearing precious metals is also the key to improve the enhancement factor and develop a variety of functions. Recently, 3D SERS substrates with z-axis spatial extension have attracted much attention due to their special structure [98]. The 3D SERS substrate has a larger total surface area, a greater number of hot spots, higher laser utilization efficiency, and greater flexibility.

#### 3.3.1. Three-Dimensional Nanocavities

Nanocavity refers to a kind of cavity structure on the nanoscale, which can confine optical or electromagnetic waves in a very small volume. When Raman molecules enter the cavity, they will produce very strong Raman signals due to these confined optical or electromagnetic waves. Such nanocavity structures are commonly used for detection at the single-molecule level [99,100]. In contrast to other structural substrates, whose optical properties are fixed after fabrication, 3D nanocavities can actively regulate the EM/CM effect by adjusting the electron cloud distribution or Fermi level of the material through temperature adjustment or electrochemical adjustment, so as to achieve a dynamic SERS signal adjustment.

**Figure 10:**
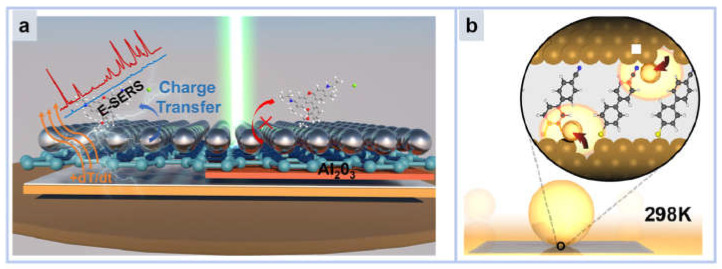
(**a**) Schematic representation of silver nanocavities regulated by temperature gradient. (**b**) Schematic representation of nanocavities formed by gold nanoparticles arranged into mirrors.

Zhang Chao et al. fabricated a silver nanocavity structure [101] using graphene as a spacer coupled to GaN. As shown in Figure 10a, the GaN film achieves EM and CM double-tuned SERS signals by generating surface spots through the temperature gradient, thereby mediating the electron density around graphene and AgNP. However, both the LSPR peak and sensitivity of this nanocavity structure are affected by temperature, and the LSPR summit shows red-shift and blue-shift phenomena during heating and cooling. The detection limit of R6G at room temperature is only 10^−8^ M, but it can be increased by an order of magnitude after heating. Although nanocavities generally require a higher temperature to achieve better sensitivity, it is not impossible to detect single molecules at room temperature.

Cloudy Carnegie et al. by using two smoothly aligned plasma modules, a nanocavity was constructed [102]. Such picocavities below 1 nm^3^ produce millions of nanospectic particle (NPoM) constructs on each sample by nanoself-assembly, as shown in Figure 9b. Each structure comprises a gold nanoparticle positioned above a flat gold film, with a molecular spacer in between, forming a plasmonic dimer due to the interaction between the nanoparticle’s electrons and the induced image charge in the film. These configurations create nanoscale gaps that focus the incoming light, allowing for high-resolution SERS analysis capable of capturing single-atom movements in real time. This nanocavity structure makes good use of the light wave in the whole three-dimensional space of the cavity, so that the SERS signal is no longer limited to the plane excitation. This nanocavity structure has a good application for single-molecule detection and observation of the mechanism of the photocatalytic process. In addition, nanogaps are also three-dimensional spatial structures similar to nanocavities, which are also two nanometer-level spacer layers that can be used for single-molecule detection of biomolecules. However, since SERS detection requires the adsorption of the molecules to be tested, such nanocavities that are difficult to fabricate need to be reused, and the desorption of already adsorbed signal molecules is also a new challenge.

**Figure 11:**
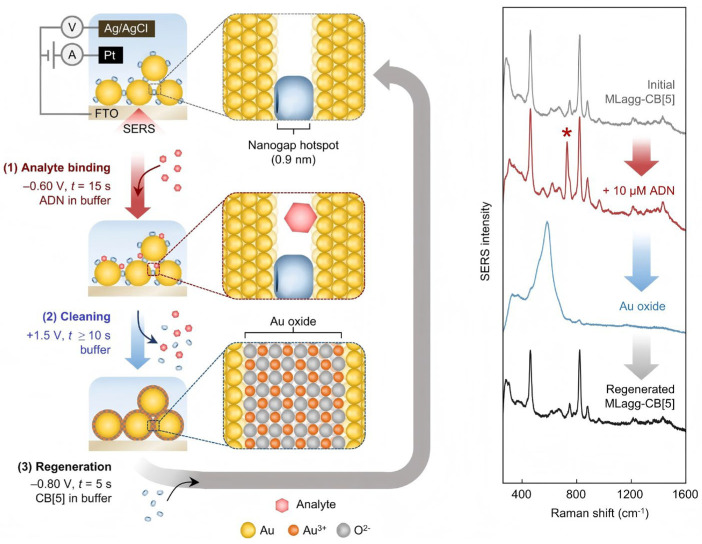
Schematic of generation of highly reproducible sub-nanometer gaps in MLagg. Adenine peak at 732 cm^−1^ is marked by asterisk [103].

Sarah May Sibug-Torres has demonstrated a low-cost scalable method [103] to fabricate thin-film SERS substrates by bottom-up self-assembly of Au NPs using rigid molecular scaffolds, cucurbit[n]uril (CB[n], n = 5–8), which generates highly reproducible sub-nanometer gap hot spots in multilayer AuNP aggregates “MLagg” (Figure 11). MLaggs are integrated into electrochemical SERS (EC-SERS) flow cells to continuously form metal oxide plugs and peel off adsorbents through electrochemical reactions. Using this cleaning and regeneration scheme, the same SERS substrates can be reused with a high level of repeatability and regeneration cycle number. The high detection level could still be maintained after 20 cycles. The SERS substrate, which is reciprocated by electrochemical reaction, meets the requirements of environmental protection and the cleaning method is quick and simple, which has broad prospects in practical application of biological detection.

Nanocavity is a highly sensitive three-dimensional substrate structure. The self-assembly method simplifies the preparation process and has an excellent performance in the application of single-molecule detection, and even the observation of some chemical reactions. However, ensuring the nanoscale cavity and controlling the entry flux of the tested substance are still the difficulties faced by this substrate in biological detection. Especially in the detection of multiple biological analytes, different individual sizes in the detection fluid may cause cavity blockage, such as CTCs and exosomes. Therefore, the application of this efficient detection substrate in biological detection needs to be further developed.

#### 3.3.2. 3D Multilayer Substrate

**Figure 12:**
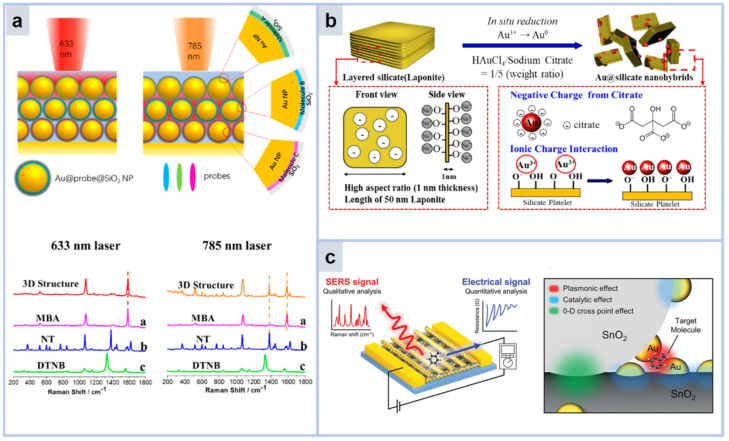
(**a**) Scheme of hot spots and Raman normalization data generated in 3D Au@probe@SiO_2_ core–shell nanostructures. Curves a b c correspond to the Raman fingerprint peaks of MBA, NT, and DTNB, respectively. [104]. (**b**) Nanohybrid Au@silicate dispersion mechanism. Uv-vis absorption of Au@silicate nanohybrids as a function of reaction time and their TEM images [106]. (**c**) Chemo-resistor and SERS multimodal sensing platform based on 3D crosspoint multi-function architecture (3D-CMA) [109].

3D multilayer substrates are usually prepared by layer-by-layer sequential assembly. The 3D multilayer substrate can increase the spatial height of plasmonic nanoparticles by depositing plasmonic nanoparticles on the inner layer. Based on Au NP, Jianfeng Li’s group reported a 3D multilayer substrate consisting of core–shell nanoparticle layers of three different Au@Probe @SiO_2_ (Figure 12a). 4-mercaptobenzoic acid, 2-naphthyl alcohol, and 5,5′-dithiobis (2-nitrobenzoic acid) were located in the top, middle, and bottom layers, respectively. By using different laser excitation wavelengths, hot spot transfer between different layers can be achieved on the Au@Probe@SiO_2_ substrate [104]. This research introduces an innovative approach to employing SERS for pinpointing the exact positions of hot spots and offers significant understanding for the manipulation and transfer of these hot spots at the nanoscale. In the case of 3D multilayer substrates, the outer layer typically has a higher probability of interacting with target molecules, while the use of the intermediate plasmonic region is restricted. Consequently, this reduces the overall efficiency of substrate utilization. Moreover, the compact arrangement of these multilayer substrates hinders laser penetration once it reaches the threshold. These drawbacks directly impact the SERS performance of multilayer substrates. To address this challenge, various open 3D multilayer substrates have been engineered, integrating microporous structures within the layers.

#### 3.3.3. 3D Microporous Substrates

3D microporous substrates are defined by their high porosity and a complex, interconnected architecture made up of intertwined nanowires, nanogrids, and nanofibers. These substrates possess substantial internal and external surface areas, offering an abundance of hotspots and localized surface plasmon regions for the capture of target molecules [105]. 3D microporous substrates inherit the excellent adsorption performance of microporous media.

The spatial mesh of nanofibers composed by electrospinning, in which noble metal nanoparticles are grown or modified, is a low-cost and rapidly fabricated 3D microporous substrate. Qiu Zhiwei’s team fabricated a novel core/shell composite nanofiber film by coaxial electrospinning. The shell consists of Au@silicate nanohybrids, Au nanoparticles (AuNPs) immobilized in silicate nanosheets by electrostatic attraction. The core of polyvinyl alcohol (PVA) offers structural integrity to the evenly distributed Au@silicate nanocomposite. This core/shell composite nanofiber film formed by random deposition has a 3D network structure. Even though the authors reduced the amount of AuNP in the nanofiber substrate, the SERS sensitivity of the nanofiber substrate was the same as that of uniaxial electrospun nanofibers. Uniaxial electrospun nanofibers have a SERS enhancement factor (EF) of 4.1 × 10^4^, while coaxial electrospun nanofibers have a SERS enhancement factor (EF) of 1.7 × 10^5^ [106]. With the help of high porosity, the analyte can be preconcentrated close to the SERS active zone.

The nanoporous metal layer entrusted by the heat-strain substrate is also a highly sensitive 3D microporous substrate. This porous metal has a large number of pore structures, which are interconnected or independent, forming complex three-dimensional networks. During thermal strain, which contains rich Raman active nanogaps generated by deformation and fracture of nanolinear metal ligaments, large electromagnetic fields are generated near the narrow nanogaps between sharp corners and edges of nanostructured noble metals.

Zhang ling et al. reported a wrinkled nanoporous gold film [107]. Thermal shrinkage of the underlying pre-strained polymer substrate (PS) can produce quasi-periodic wrinkles of the metal film, and wrinkles can turn two-dimensional planar films into three-dimensional (3D), creating more hot spots for superior SERS performance. Jin-Hyun Ham et al. reported a reusable wrinkled nanoporous silver film (w-npAgF) [108]. The signal intensity of the thermally shrunk folded porous Ag NPs is up to 8-fold higher than that of the flat Ag NPs, and the w-npAgF can be treated with gentle O_2_ and Ar plasma for SERS substrate reuse.

The substrate’s microporous architecture can significantly mitigate laser-induced thermal impacts and prevent the creation of certain carbon-based compounds from laser energy during SERS analysis. Nevertheless, it is highly challenging to fabricate 3D interconnected structural substrates by integrating microporous materials with plasmonic nanomaterials. Particularly, controlling the distribution, quantity, and dimensions of pores in three-dimensional space poses a significant difficulty. However, generating rich, stable, and controllable hotspots is the key to achieve high SERS enhancement.

#### 3.3.4. 3D Array Substrate

3D array substrates usually use a bottom-up strategy to plant materials on solid substrates to form a stereoscopic array structure. Due to the z-axis dimension of the array, the array substrate has a larger surface area and more hot spots than the planar substrate. Thus, array substrates can significantly improve the efficiency of target molecules to enter the SERS active region. Zheng et al. presented a label-free SERS multimodal sensor based on a system assembled 3D crosspoint multifunctional nanostructure (3D-CMA). 3D-CMA enables successful selective quantitative estimation of gas mixtures below the 100 ppm level through the three-dimensional integration of semiconducting SnO_2_ nano-edge frames and bifunctional gold-metal nanoparticles [105]. The closely-spaced trilaminar substrate exhibits robust near-field enhancement and efficient light-harvesting capabilities. However, the fabrication of these orderly arranged substrates typically necessitates advanced techniques like electron beam lithography, laser interference lithography, and inductively coupled plasma reactive ion etching. Moreover, the intricate fabrication process and the requirement for specialized equipment greatly hinder the mass production of these substrates. Table 1 lists SERS sensors based on nanoparticle and substrate modifications in recent years.

### 3.4. Optical Layer, Dielectric Layer Regulation Substrate

Through some optically modulated substrates, a strong and uniform magnetic field can be obtained in the process of binding the light field and the probability of collision between photons and Raman molecules can be increased. For example, effective laser utilization can be generated by using optical resonator reflection and electric field energy storage [120,121,122], or the characteristics of periodic refractive index distribution of photonic crystals can be used to modulation light waves, and effective electromagnetic field superposition and laser utilization can be provided based on photonic band gap effect [123,124,125,126].

#### 3.4.1. Photonic Crystal Substrate

**Figure 13:**
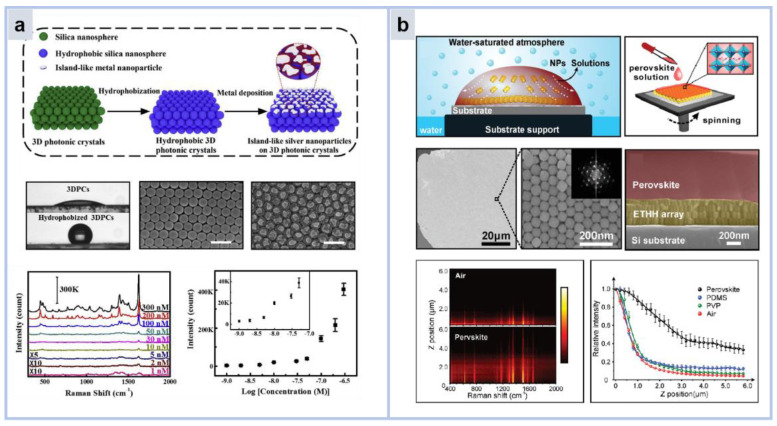
(**a**) Schematic of the fabrication process of a 3D island hybrid substrate for a 3D photonic crystal with hydrophobic Angle changes and SEM [127]. (**b**) Schematic representation of an elongated tetrahexahedral gold nanoparticle (ETHH Au NP) array and perovskite-coated ETHH NP array [128].

The plasma–dielectric hybrid substrate of Ag islands on a 3D photonic crystal by Chen et al. [127] was fabricated by magnetron sputtering of silver onto a hydrophobic silica photonic crystal without an etching process (Figure 13a). Light–matter interactions are enhanced due to the enhanced electromagnetic fields and scattering of plasmonic nanoparticles and the slow photon effect provided by photonic crystals. Significant enhancement of Raman signal can be achieved under dual action. The detection limit of adenine by the hybrid substrate reached the nM level, and the calculated enhancement factor was 1.13 × 10^7^. In addition, it is easy to implement hybrid substrate-based microchips that enable microdetection by superhydrophobic concentrations. The straightforward production and potent Raman signal amplification render Ag island-covered 3D photonic crystals as promising contenders for applications in chemical sensing, Raman mapping, and bioassay analysis.

#### 3.4.2. Dielectric Layer

Although the Raman signal intensity of noble metal nanoparticles can be improved by many orders of magnitude, this enhancement is limited to extremely short distances from the Raman active surface. Crucial for advancing the use of Raman spectroscopy in cancer and inflammatory disease diagnosis, as well as in pharmacological applications, is the capacity to identify analytes that do not engage with Raman-active surfaces. Here, Qiao et al. constructed a novel Raman enhancement system by covering an elongated tetrahexahedral gold nanoparticle array (Figure 13b) with a superficial perovskine (CH_3_N_3_PbBr_3_) film [128]. Namely, shallow enhanced Raman scattering (SLERS). Since the dielectric coefficient of perovskite is smaller than that of air. Therefore, compared with air, the attenuation of evanescent waves is suppressed in the vertical direction away from the plasma surface. Thus, the detection depth of SERS increases in the perovskite medium. The vertical penetration of SLERS was verified through the spatial distribution of analytes by Raman imaging in layer scan mode. By changing the surface permittivity of the array layer, the slow decay of the evanescent wave is controlled to slow down the attenuation of SERS signal on the z-axis. This work has research implications for label-free observation and imaging of cells and related biological applications. This work has research implications for label-free observation and imaging of cells and related biological applications. However, it is well known that perovskites are not environmentally friendly materials, and the soft bond connection mode makes their performance unstable. Therefore, more environmentally friendly, stable, and low-cost dielectric layers are a topic that needs to be studied.

#### 3.4.3. Bragg Mirror

**Figure 14:**
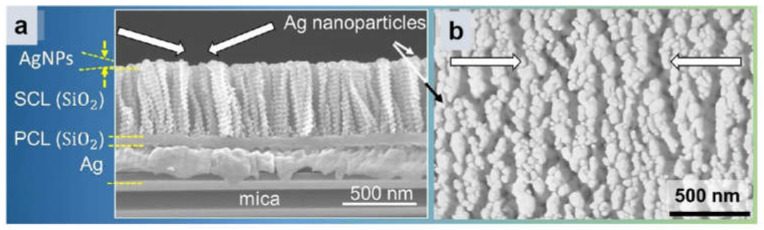
SEM micrographs of ML2 films. (**a**) Cross section; (**b**) top view [129].

The multilayer film of Bragg mirror structure is also an optical modulation substrate, and the principle of Bragg mirror operation is based on the interference phenomenon of light. When light waves propagate between two media with different refractive indices, they are reflected at the interface. If the two media are arranged alternately into multiple layers, each layer being precisely controlled at a quarter wavelength (for normal incidence), then the difference in the optical path of the reflected light at the interface between the two adjacent layers at a particular operating wavelength will be exactly half a wavelength. This optical path difference leads to a destructive interference between the reflected light, which enhances the intensity of the reflected light. For example, multilayer thin film substrates with Ag nanorod array/dielectric/mirror structure can be used to enhance the Raman scattering cross section by the interference of light. Samir Kumar et al. proposed a multilayer thin-film sheet dispersion gel [129] to prepare multilayer thin-film ML2 by continuous deposition of Ag NPs via dynamic beveling deposition (Figure 14). A thin sheet of ~10 μm was obtained by mixing the multilayer film and hydroxyethyl cellulose (HEC) gel by centrifugation, so that the optical properties of the multilayer film could be preserved. The multilayered thin-film sheet dispersion gel showed an approximately 20-fold higher SERS signal than the Ag nanorod array dispersed by gel without the multilayered thin-film structure.

The introduction of optical modulation substrates such as photonic crystals and Bragg mirrors does not directly increase the Raman scattering cross section, but the intensity of SERS is greatly improved in terms of the efficiency of excitation light utilization. In addition, Bragg mirrors and photonic crystals can locally enhance the electromagnetic field, thereby significantly improving the intensity of SERS signal. In real time, photonic crystals and Bragg mirrors are more likely to be a rigid substrate, which limits references in terms of biological work. We are looking forward to a substrate that can still maintain good optical modulation under the flexible condition, which also has a wide application prospect in the detection.

## 4. Tip Enhanced Raman Spectroscopy (TERS)

Tip-enhanced Raman spectroscopy (TERS) is an emerging branch of Raman spectroscopy, which is similar to SERS in that nanostructure plasmas will enhance and localize the incident and scattered electromagnetic fields when they are in extremely close proximity, and the principle is also based on LSPR [2]. The tips of TERS are usually SPM probes with Au/Ag coating, or fine needles formed directly using Au NPs or Ag NPs [130]. The tip radius of such probes is usually smaller than the optical diffraction limit; therefore, Raman scanning resolution in TERS is constrained by the optical diffraction limit [131]. Typically, the resolution of TERS ranges from tens of nanometers to a few nanometers. This sub-nanometer ultra-high resolution allows TERS to identify and observe single molecules and perform nanoscale scanning imaging [132,133,134,135]. Processes such as REDOX, photocatalysis, and interfacial chemical transitions are tracked with nanoscale precision [133,136]. In biological aspects, it can resolve the sequence and structure of DNA, RNA, peptides, and other biological molecules [137,138].

### 4.1. Single-Molecule Detection

As technology has evolved over the past year, TERS has moved from nanoscale precision to Angstrom precision.

**Figure 15:**
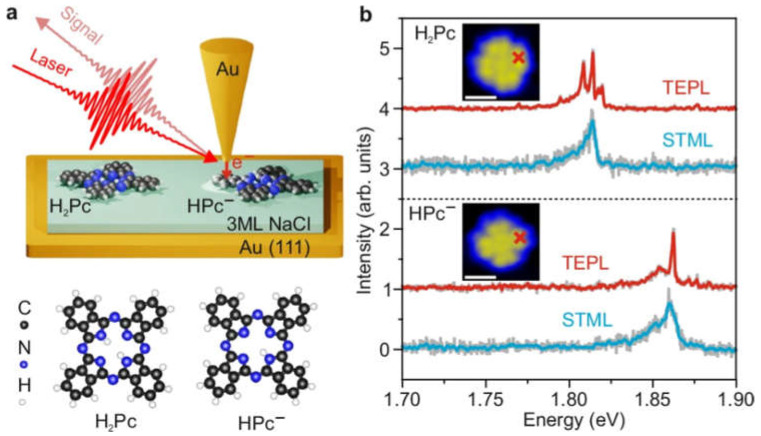
(**a**) Top panel: schematic illustration of the experimental setup for STM-induced electroluminescence (STML) and tip-enhanced photoluminescence (TEPL) measured from single molecules adsorbed on three-monolayer thick NaCl on top of Au (111). Bottom panel: the structures of the H_2_Pc and HPc^−^ molecules. (**b**) STML (blue curves) and TEPL (red curves) spectra of individual H_2_Pc and HPc^−^ molecules. Schematic representation of the detection of phthalocyanine molecules by Raman signals with laser wavelength changes [135].

This makes it possible to detect the vibrational fingerprints of individual molecules with angstrom scale spatial resolution. However, achieving controlled excitation of specific vibrational modes in a single molecule remains challenging. Luo et al. demonstrated the selective excitation and detection of vibrational modes of single deprotonated phthalocyanine molecules using resonance Raman spectroscopy under scanning tunneling microscopy [135]. The probe tip of the microscope is made of Au NPs, and the detection is achieved by selectively excitation of the wavelength of the fine-tuning laser. When the changing laser irradiates the tip and the phthalocyanine molecule, the vibrational transition between the vibrational energy levels in the base electronic state and the excited state of the phthalocyanine molecule creates resonance, resulting in rotational selectivity enhancement of the resonant Raman signal (Figure 15). Although adjusting the wavelength of the laser does not change the vibrational transition energy, it does change the energy of the Raman scattered light. Therefore, the authors used this method to explore the selective excitation of molecular vibration to control the chemical transition process of surface molecules, providing a new idea for exploring the occurrence of optical excitation in a single molecule. It also provides inspiration for the exploration of the interaction between photons and matter at the sub-molecular level.

### 4.2. Chemical Reaction Detection and Imaging Detection

TERS can accurately reflect the changes of some molecules in chemical reactions, such as ultra-high vacuum tip enhanced Raman spectroscopy (UHV-TERS), which has angstrom-level resolution and is a powerful tool for investigating site-specific chemistry and interlayer interactions in 2D materials. It has been used to characterize the atomic-level lattice strain and oxidation behavior of borene monolayers.

**Figure 16:**
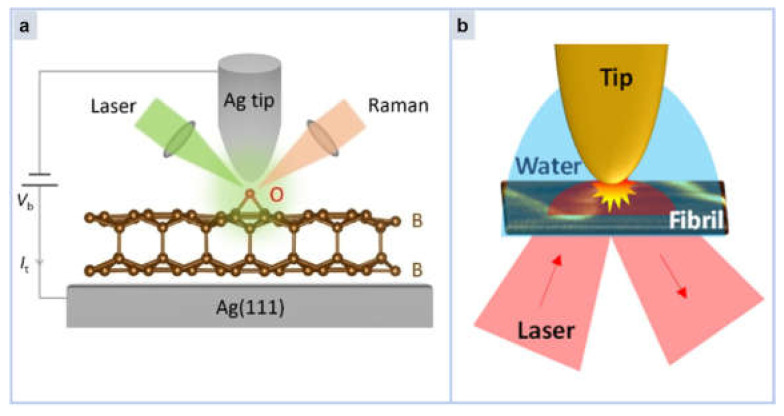
(**a**) Schematic diagram of the chemical characterization of BL borophene at the atomic level by UHV-TERS [136]. (**b**) Schematic representation of nanoscale spatial imaging of Aβ protein by TIR-TERS in water [137].

Li Linfei et al. introduced the chemical characterization of BL borophene at the atomic level using UHV-TERS [136]. The Angstrom scale spatial resolution identification of the vibrational fingerprint of BL borophene by UHV-TERS is shown in Figure 16a. The Raman spectra observed in this work are strongly correlated with the vibrations of the boron-boron bonds between layers, and the 3D lattice geometry of BL borene is verified. The authors demonstrated enhanced chemical stability of BL borene when exposed to a controlled oxidation atmosphere compared to monolayer borene. In addition to providing fundamental chemical insights into BL borophene, this work establishes that UHV-TERS are capable of exploring surface reactivity and interlayer bonding of 2D materials at the Angstrom scale, providing a powerful tool for studying chemical reaction processes at the atomic scale.

Similarly, TERS can also be imaged by spatial scanning, which often has nanoscale spatial resolution and can clearly show the state of the material to be measured. As shown in Figure 16b, Huang et al. performed nano-spatial imaging of β-amyloid protein (Aβ) in water by total internal reflection tip Raman enhanced spectroscopy (TIR-TERS) [137]. This work successfully demonstrated the main component molecules and secondary structures of Aβ, and clearly showed the nano-spatial distribution of aromatic amino acids such as tyrosine and histidine. Conclusions from TERS imaging experiments in water confirm that the structure of amyloid fibrils is not severely affected by hydration. This is the first study of TIR-TERS in liquid, which confirms the feasibility of TIR-TERS experiments in aqueous media and is more valuable for biological research than those in air or vacuum environment, opening up prospects for future biological applications.

### 4.3. Biomolecular Analysis

**Figure 17:**
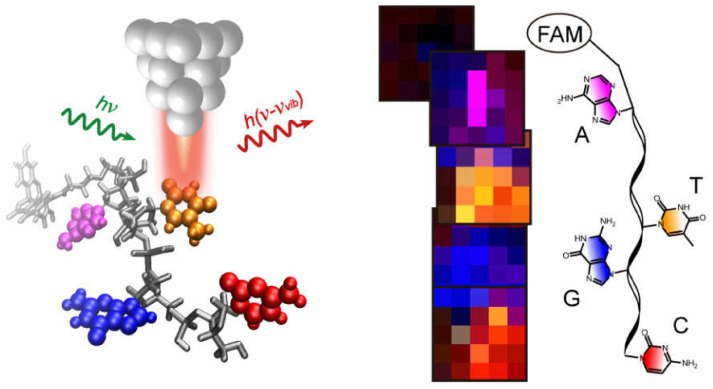
Schematic representation of nucleobase determination of DNA by TERS [138].

The resolution of the sequence and structure of biomolecules is essential for understanding the biological mechanism and function of DNA, RNA and other molecules. Single-molecule tip-enhanced Raman spectroscopy (SM-TERS) has recently been developed to identify base pairs in DNA strands without labels. Han et al. conducted a proof-of-principle exploration [138] by developing SM-TERS with sub-nanometer resolution and demonstrating a method to realize the recognition of single nucleobases in short-stranded DNA (ss-DNA) molecules as shown in Figure 17. They designed an Ag protrusion at the tip of TERS to form a needle tip. Moreover, the surface stability of ss-DNA was improved and its thermal motion was limited in liquid nitrogen environment. As long as the Raman fingerprint of this DNA is distinguishable, it can be detected and identified. Compared with other traditional methods, this method does not require labeling, enrichment, and replication, and requires very small sample volume, so it is beneficial to detect some rare biomolecules that are difficult to handle.

The advantage of TERS is its surprising resolution, which enables many single-molecule level detection and shows great promise in the dynamic analysis of chemical reactions, the identification and detection of rare biomolecules. However, the current status of TERS is too time-consuming in data measurement and analysis, and has a poor performance in application scenarios other than single-molecule analysis, especially in biological analysis. Compared with commercially available DNA sequencing technologies (such as Sanger sequencing), it is still far from practical in sequencing.

## 5. Conclusions and Outlook

This paper reviews the application progress of local field-based surface Raman enhanced (SERS) technology in the field of biosensing. SERS technology has attracted much attention due to its high sensitivity, selectivity, and ability to provide molecular fingerprinting level information. By exploiting the local surface plasmon resonance (LSPR) effect of noble metal nanostructures, SERS technology greatly enhances the Raman scattering signal, making the detection limit to the single-molecule level. This review focuses on the applications of SERS in biomolecular detection, bioimaging, and disease diagnosis, and discusses the design and optimization of different types of SERS sensors.

In the field of biosensing, SERS technology has shown great potential and advantages. Nano-label-based SERS sensors have been widely studied because of their simple preparation and low cost, especially showing high sensitivity in the detection of circulating tumor cells. The core–shell structure and MOF structure SERS sensors improve the detection stability and sensitivity by combining the characteristics of different materials. The chip-based SERS sensor provides uniform SERS active sites by uniformly arranging the nanoparticles on the substrate, which enhances the stability and reproducibility of the signal. TERS technology has shown great potential in the detection of biological single molecules due to its ultra-high resolution imaging ability.

Looking into the future, the application of SERS technology in the field of biosensing still faces some challenges and opportunities. The application of SERS technology in the field of biosensing still faces some challenges and opportunities. On the clinical side, tag detection has been an approach to overcome solution complexity and improve SERS specificity. This detection can directly identify the nano-label bound to the analyte and identify it through the Raman spectrum of the tag. However, due to the complexity of the tag detection process, and the identification and detection through SERS tags will lead to the loss of the Raman spectrum information of the original analyte. Therefore, the development trend of clinical diagnosis is still label-free detection. This rapid and complete molecular fingerprint method has great application value. The difficulty of label-free detection of biomolecules and disease markers lies in the chemical complexity of biological samples. In the case of label-free detection, SERS substrate enhances the signal of all molecules adsorbed on its surface, both detection and interference molecules. Therefore, the limitation of SERS-based biodetection lies in the low selectivity of the SERS substrate itself. Only analytes with high SERS substrate binding ability can be effectively identified and enhanced, and if such strong affinity is interference, it will lead to strong background noise and interfere with the diagnostic results. The fact is that the amount of effective analytes in disease diagnosis and clinical analysis is often not high, such as CTCs and CTDNA in cancer detection. Although the sensitivity of SERS substrate is high enough, its direct application in label-free biology is limited by a series of problems such as specificity and enrichment. In addition, the size of individual objects to be tested, such as cells and biological tissues, is usually in the micrometer level, which makes it difficult to detect the complete signal when the SERS enhancement range is in the nanometer level. Usually, SERS substrates must penetrate the biological membrane to release effective signal molecules to the substrate surface for detection.

Fortunately, with the development of materials science, nanotechnology, instrument manufacturing, data analysis, and artificial intelligence, the difficulties related to direct detection in clinical applications are gradually being overcome. SERS provides specific information at the molecular level for the identification of different diseases, including cancer, infectious diseases, etc. Combined with machine learning techniques, SERS is able to identify complex patterns for the identification, quantification, and classification of microorganisms and different diseases. Many side-stream and point-of-care devices have now been developed to cope with pandemics and have diagnostic performance for other diseases that is equal and comparable to existing commercial technologies. SERS outperforms commercial enzyme-linked immunosorbent assay (ELISA) detection kits for cancer detection, allowing for multiple analyses with very small sample sizes.

How to further improve the stability and reproducibility of SERS substrates, especially in complex biological environments, is an urgent problem to be solved. Secondly, the development of novel multifunctional SERS substrates that integrate targeting, therapeutic and diagnostic properties is of great significance for the realization of precision medicine. In addition, with the development of machine learning and artificial intelligence technology, combining SERS technology with big data analysis will help improve the accuracy and efficiency of detection. In terms of technological innovation, exploring novel nanomaterials and structures, such as 2D materials and 3D porous structures, to increase SERS active sites and improve detection sensitivity, is an important research direction. At the same time, optimizing the integration and miniaturization of SERS detection systems will help to develop portable detection equipment and expand the application of SERS technology in rapid detection in the field.

In conclusion, SERS technology has a broad application prospect in the field of biosensing. With the development of new materials, the improvement of detection technology and the improvement of data analysis ability, SERS technology is expected to play a more important role in biomedical research and clinical diagnosis in the future.

## Figures and Tables

**Table 1:** Nanoparticles, substrate, LOD, and description of SERS Sensors.

Ref.	Platform	Substrate	Material	Target	Description	LOD
[33]	Single crystal	Hydrogel	Ag NWs@RC	Nano plastic	Ag NWs@RC was used for the detection of nanoplastics in water	0.1 mg/mL
[110]	Core shell	Si	Si/TiO_2_@Ag	R6G	EM and CT synergistic SERS enhancement in Si/TiO_2_/Ag heterostructures via local interfacial effect	0.1 pM
[111]	Core shell	-	Au NPs@MnO_2_	GSH	MnO_2_-coated Au nanoparticles advance SERS detection of cellular glutathione	1 μM
[112]	Core shell	GO	pAu/G/PBA	*S. aureus*	Detecting and inactivating Salmonella and Staphylococcus with a background-free SERS chip	10 CFU/mL
[113]	MOF	-	Fe_3_O_4_@Ag@COF	MTD	Molecules were enriched using a magnetic metal shell and detected by SERS	0.1 nM
[114]	MOF	-	Au NPs@ZIF-8	VOC	Detection of volatile organic compound gas by a scalable plasma gas sensor	1 nM
[115]	MOF		Au NPs@MIL-100	*V. parahaemolyticus*	Colorimetric SERS dual-mode was used to detect Vibrio parahaemolyticus	9 cfu/mL
[116]	Single crystal	NiCoLDH	Au-NPs/NiCoLDH/CC	FP	Au NPs/NiCoLDH flexible SERS substrate for real-time detection of fipronil	3.78 nM
[117]	Single crystal	AgNS600	AgNS600	glucose	A simple physical scratch fabrication of SERS substrate	0.5 aM
[118]	Array	Silicon wafer	Ag NPs	MTO MB	Self-assembled superhydrophobic silver film with interfacial assembly	8 × 10^−10^ M 3 × 10^−8^ M
[119]	Array	PDMS	Au NSs	estrogen	3D micro/nano plasma substrate binding azotization based on Au NSs monolayer films	10^−11^ mg/mL
